# Absence of truncating *BRIP1* mutations in chromosome 17q-linked hereditary prostate cancer families

**DOI:** 10.1038/sj.bjc.6605433

**Published:** 2009-11-24

**Authors:** A M Ray, K A Zuhlke, G R Johnson, A M Levin, J A Douglas, E M Lange, K A Cooney

**Affiliations:** 1 Department of Internal Medicine, University of Michigan Medical School, Ann Arbor, MI, USA; 2 Department of Human Genetics, University of Michigan Medical School, Ann Arbor, MI, USA; 3 Department of Genetics, University of North Carolina, Chapel Hill, NC, USA; 4 Department of Urology, University of Michigan Medical School, Ann Arbor, MI, USA

**Keywords:** prostate cancer, *BRIP1*, genetic variation

## Abstract

**Background:**

In a genome-wide scan (GWS) of 175 multiplex prostate cancer (PCa) families from the University of Michigan Prostate Cancer Genetics Project (PCGP), linkage was observed to markers on chromosome 17q21–24, a region that includes two breast cancer susceptibility genes, *BRCA1* and *BRIP1*. *BRIP1* is a Fanconi anaemia gene (*FANCJ*) that interacts with the BRCT domain of BRCA1 and has a role in DNA damage repair. Protein truncating mutations in *BRIP1* have been identified in hereditary breast and ovarian cancer families, and a recent report suggested that a recurrent truncating mutation (R798X) may have a role in PCa susceptibility.

**Methods:**

We examined the role of *BRIP1* mutations in hereditary PCa through sequence analysis of 94 individuals from PCGP families showing linkage to 17q.

**Results:**

A total of 24 single-nucleotide polymorphisms, including 7 missense variants but no protein truncating mutations, were observed.

**Conclusion:**

The data presented here suggest that *BRIP1* truncating mutations are uncommon in PCa cases and do not account for the linkage to chromosome 17q observed in our GWS. Additional investigation is needed to determine the significance, if any, of the observed *BRIP1* missense variants in hereditary PCa.

Prostate cancer (PCa) is the most commonly diagnosed cancer and the second leading cause of cancer death among American men (Jemal *et al*, 2009). In addition to age and ancestry, family history is a leading risk factor for developing PCa, suggesting that germline genetic variation has a role in PCa development. However, the number and identities of the genes involved in hereditary PCa are still largely unknown.

There is some evidence to suggest that inherited mutations in breast cancer (BrCa) and ovarian cancer (OvCa) risk genes *BRCA1* and *BRCA2* also increase the risk of PCa. Several studies have shown that carriers of deleterious, germline mutations in *BRCA2* are at an increased risk of developing PCa, especially early-onset PCa (Risch *et al*, 2001; Thompson and Easton, 2002; Edwards *et al*, 2003; Kirchhoff *et al*, 2004; Agalliu *et al*, 2007). In addition, we recently found that two common *BRCA1* single-nucleotide polymorphisms (SNPs) are associated with familial and early-onset PCa (Douglas *et al*, 2007).

*BRIP1*, located at 17q22, is a Fanconi anaemia gene (*FANCJ*) (Litman *et al*, 2005) that directly interacts with the BRCT domain of BRCA1 (Yu *et al*, 2003) and has a role in DNA damage repair (Bridge *et al*, 2005; Litman *et al*, 2005). *BRIP1-*deficient cell lines are overly sensitive to DNA crosslinkers cisplatin and mitomycin C (Bridge *et al*, 2005; Litman *et al*, 2005), arrest in the S-phase of the cell cycle, and exhibit increased chromosomal instability (Kumaraswamy and Shiekhattar, 2007). Recently, *BRIP1* has been identified as a BrCa susceptibility gene. Seal *et al* (2006) found that carriers of truncating *BRIP1* mutations have a relative risk of BrCa of 2.0. The functional relationship between *BRIP1* and *BRCA1* and their roles in hereditary BrCa make *BRIP1* a biologically plausible candidate PCa susceptibility gene.

Prostate cancer linkage studies have identified several areas of the genome that may harbour susceptibility loci, although signals have often been difficult to reproduce between research teams (Schaid, 2004). The University of Michigan Prostate Cancer Genetics Project (PCGP) was the first group to report PCa linkage to chromosome 17q markers (Lange *et al*, 2003). Chromosome 17q linkage has subsequently turned out to be one of the strongest (Lange *et al*, 2007) and most reproducible (Gillanders *et al*, 2004; Xu *et al*, 2005) linkage signals for hereditary PCa. *BRIP1* is ∼20 cM downstream from *BRCA1* and both are contained near or within our linkage signal. Previous research from our laboratory failed to identify deleterious BRCA1 truncating mutations in chromosome 17-linked families (Zuhlke *et al*, 2004). In this study, we examine the role of germline *BRIP1* variation in hereditary PCa, focusing on families with linkage evidence to 17q markers.

## Methods

### Patient selection

A genome-wide linkage scan was previously performed on 175 families from the PCGP. To be eligible for the scan, families had to meet one of the following criteria: (1) three or more individuals diagnosed with PCa at any age in ⩾3 generations or (2) two or more individuals diagnosed with PCa before the age of 55 years. The most significant evidence for linkage was observed at ∼60 cM on chromosome 17, with 95 families showing some evidence of linkage (non-parametric linkage score >0 at 60.1 cM on chromosome 17, which is the linkage peak from our first genome-wide scan (GWS) (Lange *et al*, 2003)). One individual was selected from each of 94 linked families and was included in this analysis, including 88 men with PCa and 6 women with BrCa. The selected men were either the youngest PCa case in the family (with DNA available) or an older case in a first- or second-degree relationship with a woman with BrCa. The women selected were all first-degree relatives of at least one man with PCa. The number of BrCa and OvCa cases in each family, as reported in family history surveys, was calculated. PCa cases were confirmed by medical record when possible.

### Sequencing

All *BRIP1* exons and intron–exon boundaries were amplified by PCR and directly sequenced in each individual. PCR conditions and primer sequences are available on request. Sequencing reactions were performed using Big Dye Terminator v1.1 chemistries (Applied Biosystems, Foster City, CA, USA). Products were then analysed using an ABI 3100 Genetic Analyzer (Applied Biosystems). Sequences were screened for variants using Mutation Surveyor v2.61 software (SoftGenetics, State College, PA, USA).

## Results

Table 1 shows the clinical characteristics of the 94 PCGP families with linkage to chromosome 17 markers. Of the families, 83 were Caucasian, 10 were African American, and 1 was Asian American. There were an average of 4.1 confirmed PCa cases per family, and the average age of diagnosis of confirmed PCa cases across families was 63.4 years. In all, 37 families had at least 1 BrCa case and 8 families had at least 1 OvCa case. Five families had both BrCa and OvCa cases. We used the software, Merlin (http://www.sph.umich.edu/csg/abecasis/Merlin/index.html), to calculate the Kong and Cox maximum LOD score on the basis of the exponential model and ‘pairs’ non-parametric allele-sharing statistic at the *BRIP1* locus for the families of the 94 patients included in the sequence analysis (Whittemore and Halpern, 1994; Kong and Cox, 1997; Abecasis *et al*, 2002). The LOD score was 8.26 in these families at the *BRIP1* locus.

The 94 individuals sequenced here included 88 men with PCa and 6 women with BrCa. The PCa cases had a median age of diagnosis of 55.5 years, and BrCa cases had a median age of diagnosis of 61.5 years (Table 2). The median pre-diagnosis PSA of PCa cases was 6.15 ng ml^−1^. The majority of men with PCa were treated surgically, and ∼78% had localised disease at diagnosis.

Overall, no truncating mutations were observed, but a total of 24 SNPs were identified in the sequenced regions of the *BRIP1* gene (Table 3). In total, 11 of these SNPs were located in coding regions, including 7 non-synonymous SNPs, 5 SNPs in *BRIP1* untranslated regions, and 8 intronic SNPs. Six of the seven non-synonymous SNPs were observed in only one to two individuals. However, the minor allele of S919P was present in 55 of the 94 individuals and had an allele frequency of ∼37%. Overall, none of the individuals was invariant at all 24 SNPs. A total of 59 individuals had at least one missense variant. One individual was heterozygous for three missense variants.

Further analyses were conducted to evaluate the potential function of observed variants. All missense variants were analysed using SIFT (http://sift.jcvi.org/) and PolyPhen (http://genetics.bwh.harvard.edu/pph/) (Ramensky *et al*, 2002; Ng and Henikoff, 2006), which are computer programmes that use structural and phylogenetic data to predict the functional impact of amino-acid changes. Two SNPs, R264W and R419W, were predicted to be deleterious by both programmes. These SNPs, which were each observed in only one individual, were typed in additional family members. In one family, R264W accounted for two out of three PCa cases and was not present in the unaffected brother of the proband, who was the only unaffected male with DNA available for analysis. In the second family, R419W was present in both the proband and his affected brother; DNA was not available for any other members of this family.

## Discussion

The location of *BRIP1* near or within the strongest linkage signal identified in a GWS of hereditary PCa families, along with its functional interaction with *BRCA1* and its role in BrCa susceptibility, led us to investigate the possibility that *BRIP1* is also a PCa susceptibility gene. We screened individuals from families that were highly enriched for both hereditary PCa and chromosome 17 linkage. No obviously deleterious, truncating mutations were detected in the 94 unrelated individuals sequenced from chromosome 17-linked families. However, multiple SNPs, including 7 missense variants, were identified. Although the precise function of these SNPs is unknown, both R264W and R419W were predicted to be deleterious by SIFT and PolyPhen (Ramensky *et al*, 2002; Ng and Henikoff, 2006).

Truncating *BRIP1* mutations have been clearly implicated as cancer susceptibility alleles. Seal *et al* (2006) identified five different truncating *BRIP1* mutations in nine BrCa cases from hereditary BrCa families who had tested negative for *BRCA1* and *BRCA2* mutations, and reported a relative risk of BrCa of 2.0 for mutation carriers. Additional studies by De Nicolo *et al* showed that a recombinant protein containing the novel *BRIP1* truncating mutation (2992–2995delAAGA) had decreased protein stability and diminished ability to interact with *BRCA1*. Further experiments using BrCa tissue from a patient carrying the same exon 20 four base-pair deletion confirmed that there was loss of the wild-type allele in tumour cells, consistent with the model of a classical tumour-suppressor gene (De Nicolo *et al*, 2008). It has also been shown that bi-allelic, truncating mutations in *BRIP1* cause Fanconi anaemia complementation group J (Litman *et al*, 2005).

To date, no study has definitively shown that *BRIP1* variants, other than truncating mutations, have a role in susceptibility to any disease. Sigurdson *et al* (2004) found that women who were homozygous for the serine allele at S919P had a relative risk of developing BrCa of ∼7 before the age of 50 years compared with homozygotes for the proline allele. However, this result was not statistically significant after adjustment for multiple testing. Several subsequent studies have also found no association between S919P genotype and BrCa risk (Garcia-Closas *et al*, 2006; Vahteristo *et al*, 2006; Frank *et al*, 2007). Recently, two *BRIP1* tagged SNPs were found to be associated with OvCa risk in case–control samples from the United Kingdom, Denmark, and the United States, but this association was weak and has not been validated (Song *et al*, 2007).

There is only one report to date that specifically examines the potential contribution of *BRIP1* variants to PCa. Kote-Jarai *et al* (2009) sequenced DNA from the youngest PCa case from each of 192 British multiplex PCa families. They identified a truncating mutation (R798X) in only 1 of the 192 families, and this mutation failed to completely segregate with PCa cases in this family. Sequence analysis identified an additional five non-synonymous variants, including only one variant described in our report (S919P) that was detected in 43% of cases and 46% of controls (*N*=2081). These authors concluded that truncating *BRIP1* mutations may rarely contribute to PCa susceptibility. Although we only sequenced cases from 94 families, compared with 192 in the report by Kote-Jarai, our strategy of selecting chromosome 17-linked families should have increased the likelihood of detecting *BRIP1* mutations if they segregated with PCa within these families. The fact that we did not detect any obvious deleterious mutations suggests that *BRIP1* mutations are unlikely to contribute to hereditary PCa.

In conclusion, our data suggest that *BRIP1* truncating mutations are not common PCa susceptibility alleles and do not account for the chromosome 17 linkage observed in our GWS. The potential role of the coding and non-coding *BRIP1* SNPs identified in this report remains unclear, as it is possible that a more common genetic variation contributes to PCa risk. For example, Kote-Jarai *et al* found significant PCa associations with two SNPs in intron 6 of *BRIP1*, which are correlated with at least one of the common SNPs identified here (rs4988340; based on the HapMap CEU sample, the pairwise *r*^2^=0.89 and 0.78 with rs6505074 and rs8076727, respectively). It is possible that the chromosome 17 linkage observed in multiplex PCGP families is because of mutations in a nearby gene, and the more common *BRIP1* variations observed by us and others may have more subtle influences on disease susceptibility. Therefore, follow-up association studies should be considered to assess the possible contribution of these variants to PCa risk.

## Figures and Tables

**Table 1:** Characteristics of chromosome 17-linked families (*n*=94)

	***N* (%) or mean (range)**
*Ancestry*	
Caucasian	83 (88)
African American	10 (11)
Asian American	1 (1)
	
*Number of PCa cases per family*	
Confirmed	4.1
Total	4.3
Mean age at diagnosis of confirmed PCa cases	63.7 (45.7–78.3)
	
*Number of families with BrCa cases*	
0	57 (61)
1–2	31 (33)
>2	6 (6)
	
*Number of families with OvCa cases*	
0	86 (92)
1–2	8 (9)
>2	0 (0)

Abbreviations: BrCa=breast cancer; OvCa=ovarian cancer; PCa=prostate cancer.

**Table 2:** Characteristics of sequenced individuals

	***N* (%) or median (interquartile range)**
*Sex*	
Male – PCa cases	88 (94)
Female – BrCa cases	6 (6)
	
*BrCa cases*	
Age at diagnosis	61.5 (50.25–63.75)
	
*PCa cases*	
Age at diagnosis	55.5 (50–60)
Pre-diagnosis PSA (ng ml^−1^)	6.15 (3.83–9.38)
Surgery (% yes)	69 (78.4)
Gleason scores^a^	
<7	39 (47)
7	39( 47)
>7	5 (6)
	
*Stage* a, b	
Localised	63 (78)
Locally advanced	14 (17)
Metastatic	4 (5)

Abbreviations: BrCa=breast cancer; PCa=prostate cancer; PSA=prostate-specific antigen.

a Columns do not add up to 88 due to missing data.

b Localised=T1 or T2, N0 and M0 or Pre-Dx PSA <20 ng ml^−1^; locally advanced=T3 or T4, N0 and M0 or Pre-Dx PSA >20 ng ml^−1^ but <100 ng ml^−1^; metastatic=N1 or M1 or Pre-Dx PSA >100 ng ml^−1^.

**Table 3:** Germline *BRIP1* mutations identified in hereditary PCa families

	**dbSNP ID no.**	**Location**	**Minor allele frequency**	**Number of individuals**	**Ancestry**
*Missense mutations*					
R106C		Exon 3	0.005	1	Caucasian
V193I	rs4988346	Exon 6	0.011	2	Caucasian, African American
L195P	rs4988347	Exon 6	0.005	1	Caucasian
R264W	rs28997569	Exon 7	0.005	1	Caucasian
R419W		Exon 9	0.005	1	Caucasian
T686A		Exon 14	0.005	1	Caucasian
S919P	rs4986764	Exon 19	0.373	55	Caucasian, African American, Asian American
					
*Synonymous mutations*					
L692 L		Exon 14	0.005	1	Caucasian
R762R		Exon 16	0.005	1	African American
E879E	rs4986765	Exon 19	0.282	41	Caucasian, African American, Asian American
Y1137Y	rs4986763	Exon 20	0.378	56	Caucasian, African American, Asian American
					
*UTR mutations*					
15 C>T		5′-UTR	0.005	1	Caucasian
4019 A>G		3′-UTR	0.011	2	Caucasian
4050 C>T	rs1978111	3′-UTR	0.388	56	Caucasian, African American, Asian American
4063 G>C		3′-UTR	0.005	1	African American
4375 T>C	rs7213430	3′-UTR	0.367	55	Caucasian, African American, Asian American
					
*Intronic mutations*					
IVS1+12 C>T	rs4988340	Intron 1	0.271	1	Caucasian
IVS1-3 T>C		Intron 1	0.005	1	Caucasian
IVS2+15 G>A		Intron 2	0.005	1	African American
IVS2-18 A>C	rs2138005	Intron 2	0.005	1	African American
IVS4+78 A>G		Intron 4	0.005	1	African American
IVS14+7 C>T		Intron 14	0.005	1	Caucasian
IVS17+80 A>G		Intron 17	0.032	5	African American
IVS19+43 A>T	rs4988357	Intron 19	0.335	58	Caucasian, African American

Abbreviations: PCa=prostate cancer; SNP=single-nucleotide polymorphism; UTR=untranslated region.
